# Orbital Swelling Due to Forceful Sneezing: Is It Possible?

**DOI:** 10.7759/cureus.77266

**Published:** 2025-01-11

**Authors:** Mashhood U Qazi

**Affiliations:** 1 Emergency Department, Sheikh Tahnoon Bin Mohammed Medical City, Al Ain, ARE

**Keywords:** emphysema subcutaneous, forceful, orbital fracture, orbital swelling, sneezing

## Abstract

A 28-year-old male patient presented to the emergency department with a sudden onset of unilateral orbital swelling associated with pain immediately after he had an episode of forceful sneezing. There was no history of trauma. Physical examination revealed obvious right orbital swelling. The patient had difficulty opening the right eye; however, the eye movements were within normal limits. Visual acuity was intact. In the absence of trauma, orbital cellulitis was considered as the differential diagnosis. However, computed tomography (CT) of the orbit showed a fracture of the medial wall of the right orbit with air squeezing into the nearby right orbit and large subcutaneous periorbital subcutaneous emphysema. The patient was treated conservatively with antibiotics and admitted to the hospital for five days. Subsequently, he made a full recovery with resolutions of all symptoms and was discharged home.

## Introduction

Emergency department physicians often see patients who present with orbital swellings. Unilateral orbital swellings can have a wide range of differential diagnoses. It can range from physical trauma to the orbit to non-traumatic causes. Trauma and sinus diseases are the most common causes of orbital swelling compared to non-traumatic causes, which are rare [1]. Rare presentations are the cases that present to the emergency department, and if misdiagnosed, can result in significant morbidity and mortality [2]. Blunt facial trauma resulting in fracture of the ethmoidal, frontal, or maxillary sinus usually results in orbital swelling and emphysema [3]. Orbital cellulitis and orbital emphysema can both have similar presentations in a patient having eye swelling since their presentations mimic each other, thus posing a challenge to the treating physicians. Therefore, it is important to take a comprehensive history and perform a thorough physical examination. Usually, orbital emphysema not related to trauma is a self-limiting condition and can be effectively treated conservatively [4].

Herein, we present an unusual presentation of unilateral orbital swelling that was presented to the emergency department.

## Case presentation

A 28-year-old male patient arrived at the emergency department with a sudden onset of unilateral orbital swelling, accompanied by pain, following an episode of forceful sneezing. There was no history of trauma. There was no prior medical history. 

Physical examination revealed that there was obvious right orbital swelling with the presence of surgical emphysema. There were no lacerations or signs of bruising. Eye movements were within normal limits, and pupils were equal, round, and reactive to light and accommodation. Visual acuity in both eyes was tested and was found to be intact. However, the patient had difficulty opening the right eye. Crepitus was noted on palpation of the right eye.

**Figure 1:**
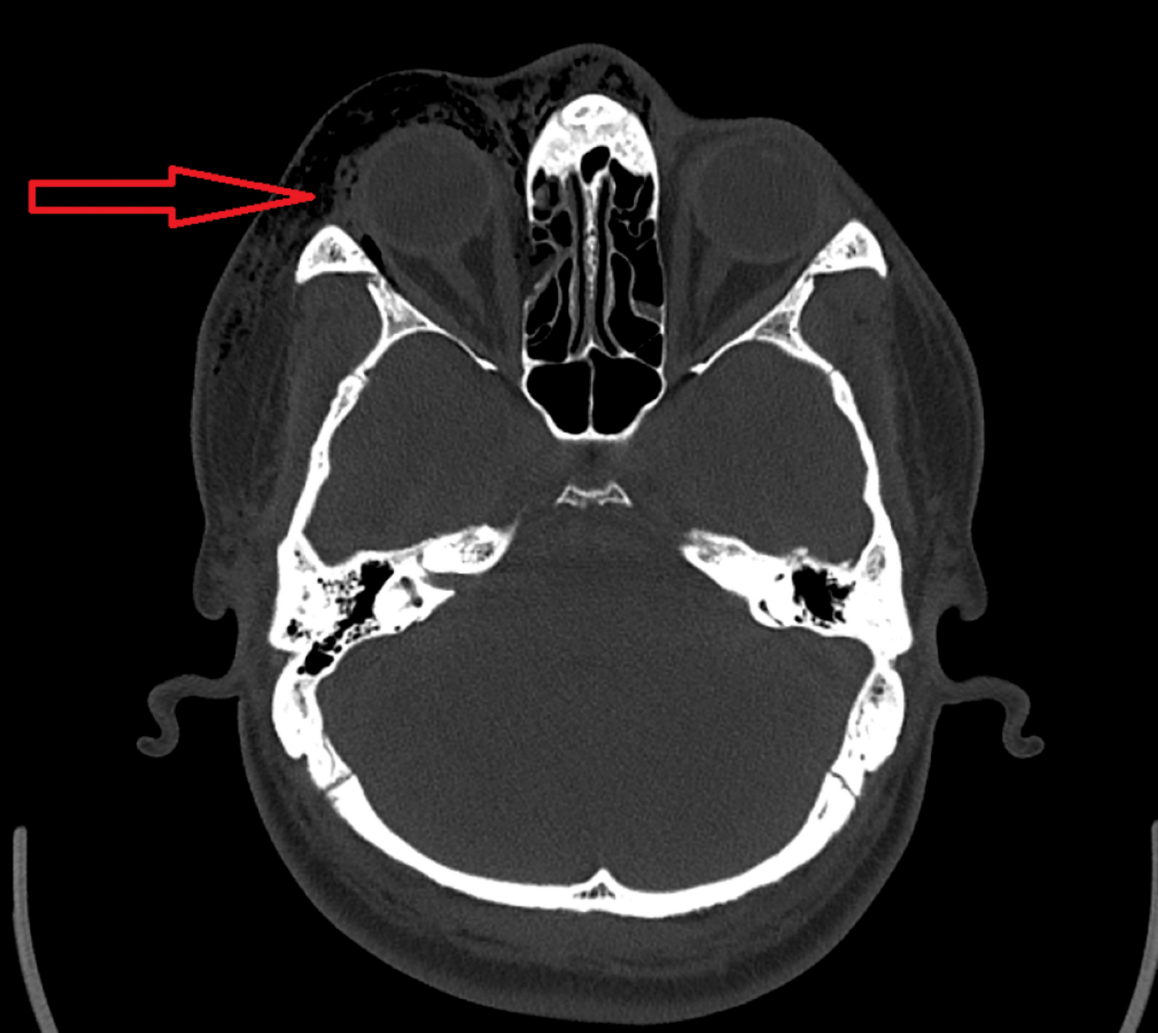
Axial CT view demonstrating right orbital subcutaneous emphysema as pointed out by the red arrow

**Figure 2:**
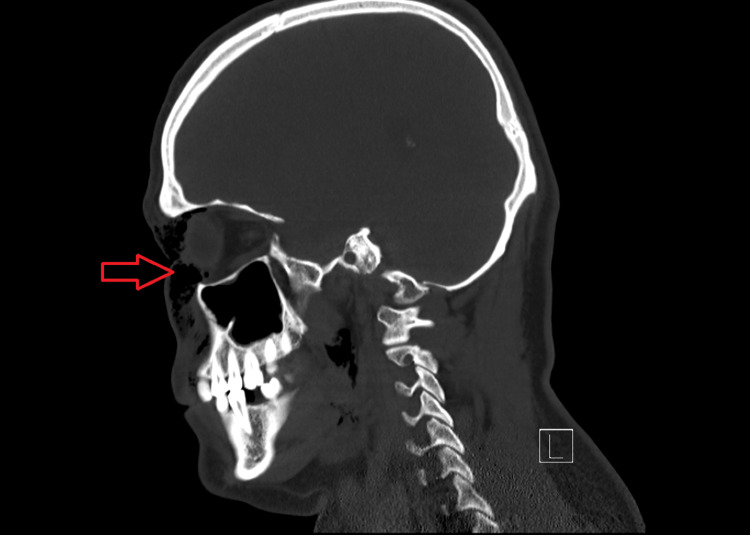
Sagittal CT view showing orbital subcutaneous emphysema

**Figure 3:**
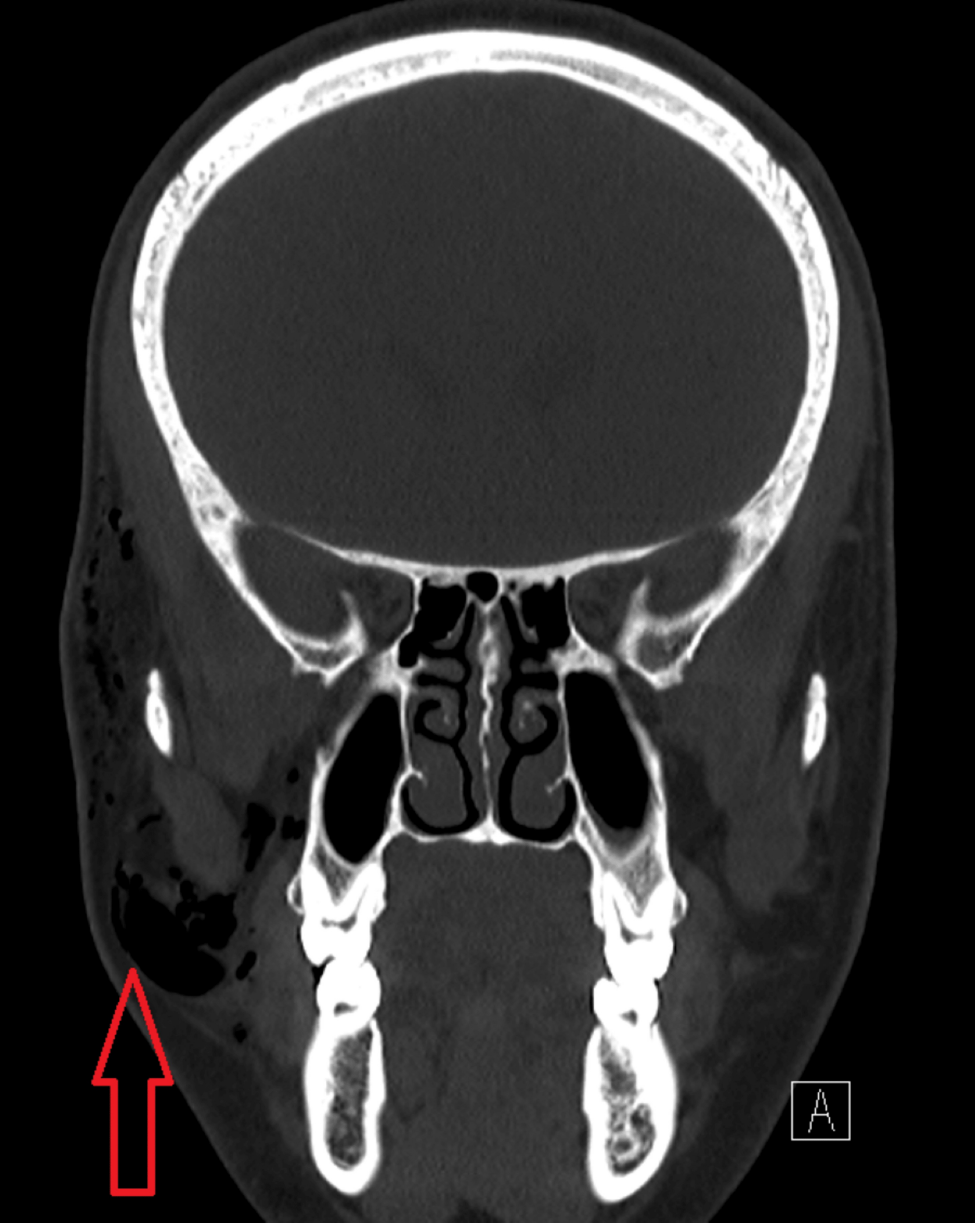
Coronal CT view showing the extent of right orbital subcutaneous emphysema as pointed out by the red arrow

Computer tomography (CT) showed a fracture of the medial wall of the right orbit with air squeezing into the nearby right orbit and large subcutaneous periorbital subcutaneous emphysema (Figure 1, 2, 3).

The patient was treated conservatively with intravenous antibiotics, co-amoxiclav, and admitted to the hospital for five days. The patient made a good recovery and was discharged home. The patient was subsequently seen in follow-up in the outpatient maxillofacial clinic in two weeks' time and was noted to have made a full recovery.

## Discussion

In most of the studies, patients who had developed orbital emphysema after sneezing had a history of old periorbital trauma or surgery [1]. There has been limited literature available regarding orbital emphysema resulting from forceful sneezing. In the Jawaid study [2], a patient presented with orbital swelling after forceful nose blowing. Kocak's study [5] mentioned a case of a 30-year-old man who developed left orbital crepitus after sneezing. 

It has been well-established that physical trauma can result in orbital emphysema. Positive airway ventilation such as continuous positive airway pressure (CPAP) can also result in orbital swelling secondary to mucosal irritation and orbital floor thinning as mentioned in the study by Komro [6]. 

With the orbit, the medial wall of the orbit is the most common place for orbital fractures [7]. In another study by Ariyoshi [8], a patient presented with orbital swelling and emphysema that had resulted from forceful nose blowing. A case report by Sen [9] mentioned a case of orbital emphysema that developed after sneezing. Treatment of non-traumatic orbital emphysema is conservative and usually involves observation and antibiotics.

## Conclusions

Without physical trauma, forceful sneezing can result in orbital fracture causing unilateral orbital swelling. Upon reviewing such patients, instructions should be given that should include advice against sneezing or nose blowing, diving, and flying since pressurized air can enter the nasal cavity.
